# Low-dose CT with adaptive statistical iterative reconstruction for evaluation of urinary stone

**DOI:** 10.18632/oncotarget.25047

**Published:** 2018-04-13

**Authors:** Xiaohu Li, Hongmin Shu, Yifei Zhang, Xiaoshu Li, Jian Song, Junhua Du, Yinfeng Qian, Bin Liu, Yongqiang Yu

**Affiliations:** ^1^ Department of Radiology, The First Affiliated Hospital of Anhui Medical University, Hefei, China; ^2^ Department of Urology, The First Affiliated Hospital of Anhui Medical University, Hefei, China

**Keywords:** urinary stone, low dose, adaptive statistical iterative reconstruction, computed tomography

## Abstract

**Purpose:**

To prospectively determine the diagnostic performance of low-dose CT (LDCT) with adaptive statistical iterative reconstruction (ASIR) technique for the detection of urinary stone disease.

**Results:**

The average DLP and ED was 408.16 ± 119.04 mGy and 6.12 ± 1.79 mSv in CDCT, and 138.19 ± 76.87 mGy and 2.07 ± 1.15 mSv in LDCT, respectively. The dose reduction rate of LDCT was nearly 66.1% for both DLP and ED (*P* < 0.05). LDCT–80% ASIR images showed great image quality (mean score = 4.09), which was similar to CDCT-FBP images (mean score = 4.17) (*P* > 0.05), but higher than LDCT-FBP images (mean score = 2.77) (*P* < 0.05).

**Materials and Methods:**

70 consetutive patients with clinically suspected urolithiasis underwent non-enhanced CT. Followed by both conventional-dose CT (CDCT) and low-dose CT (LDCT) scans. Automatic tube current modulation (ATCM) scanning was used, with a noise index setting of 13 in CDCT and 25 in LDCT. Reconstructions were performed with filtered back projection (FBP) and different settings of adaptive statistical iterative reconstruction [ASIR(40%, 60%, 80%)]. Urinary calculi (size, location, number), image quality (scale 1–5), image noise (scale 1–3) and diagnostic confidence levels (scale 1–3) were evaluated and measured by two radiologists independently. Radiation dose was recorded by calculating dose length product (DLP) and effective dose (ED). Statistical analyses included Mann-Whitney *U* test and Paired *t* tests.

**Conclusions:**

LDCT with ASIR can reduce the radiation dose while maintain relatively high image quality in the diagnosis of urinary stone diseases.

## INTRODUCTION

Urolithiasis is one of the most common disorders of urinary tract. Recent studies have suggested an increasing incidence of urolithiasis and recurrence after the first episode, especially among children and young people [1, 2]. Imaging is important for the diagnosis of acute and chronic urinary stone disease. Conventional abdominal radiology, intravenous urography and renal ultrasound (US) were usually used for the assessment of urinary stones, while about 34% of ureteral calculi, especially the X-ray negative calculi, can not be found by abdominal radiology [3]. Although intravenous urography was used for diagnosing urinary stones, the additional requirement of intravenous contrast media can cause serious complications, such as renal toxicity and acute allergic reaction. In adults, Unenhanced Multidetector computed tomography (CT) has a high sensitivity (95%–96%) and specificity (97%–100%) for the diagnosis of urinary stones, which was higher than intravenous urography or KUB [4, 5]. However, with respect to radiation dose, Unenhanced CT has a higher risk of radiation hazard than IVU or KUB, which might be a main limitation of this examination.

Exposure to small amounts of radiation can cause cancer, especially in younger patients [6]. In recent years, the medical workers and the public have paid more attention to the relationship between cancer and radiation. Reduction of the radiation dose is now essential for minimizing the concerns about CT for both patients and physicians. Reduced radiation dose during the evaluation of urinary stone is one of the most interests in the field of urology, since low-dose CT (LDCT) is a best method in the evaluation of urinary calculi.

Given the fact that urinary stone is chronic in nature and requires repeat imaging, commonly using CDCT, there may be a substantial radiation dose during an individual's lifetime. Thus, there is a need for an accurate diagnostic imaging tool with minimal radiation exposure. The high contrast between stones and the surrounding soft tissue should make it possible to substantially reduce the radiation dose without affecting diagnostic accuracy. A meta analysis shows the radiation dose for urinary stone CT acquisitions can be safely reduced below 3 mSv without affecting the diagnostic accuracy of stone detection [7]. Nowadays, the American Urological Association provides no clear recommendation [8], However the Current guidelines of the the American College of Radiology as well as the the European Association of Urology advise using low-dose CT in patients with acute disease and suspicion of urinary stone [9, 10].

Some investigators [11–13] showed that during the range of 0.7–4.2 mSv with the use of LDCT, the CT radiation dose has been significantly decreased. However, LDCT introduces an additional noise on images reconstructed with filtered back projection (FBP) technique, which affects the image quality and the radiologists' confidence during the diagnostic procedure. Recent advances in imaging technology have greatly provided a chance to decrease image noise accompanied by radiation dose reduction in CT examination. To improve image quality, some reconstruction techniques have also been introduced, such as adaptive statistical iterative reconstruction (ASIR), which can reduce image noise in the scanning image acquisition [14].

In this study, we prospectively determined the diagnostic performance of LDCT with ASIR technique for the detection of urinary stone disease. We hypothesized that this method can reduce radiation dose while maintain clinical application value.

## RESULTS

### Demographic characteristics

A total of 70 patients were finally enrolled in this prospectively study and were scanned using both conventional CT (CDCT) and low-dose CT (LDCT). For the 70 patients, 41 males and 29 females, with a mean age of 50.3 ± 13.1 years, ranging from 21–77 years. The patients' mean BMI was 24.24 kg/m^2^, and the range was 15.60–33.81 kg/m^2^.

### CT findings

In total 352 stones were present in 70 patients with CDCT. Of the 352 stones, 318 were in the kidney, 34 stones were located in the ureter (15 were upper, 9 were middle, and 10 were lower), There was no stone in the bladder. The size of the stones was was smaller than 3 mm (76/352, 22%), 3–10 mm (174/352, 49%), or larger than 10 mm (102/352, 29%).

**Figure 1:**
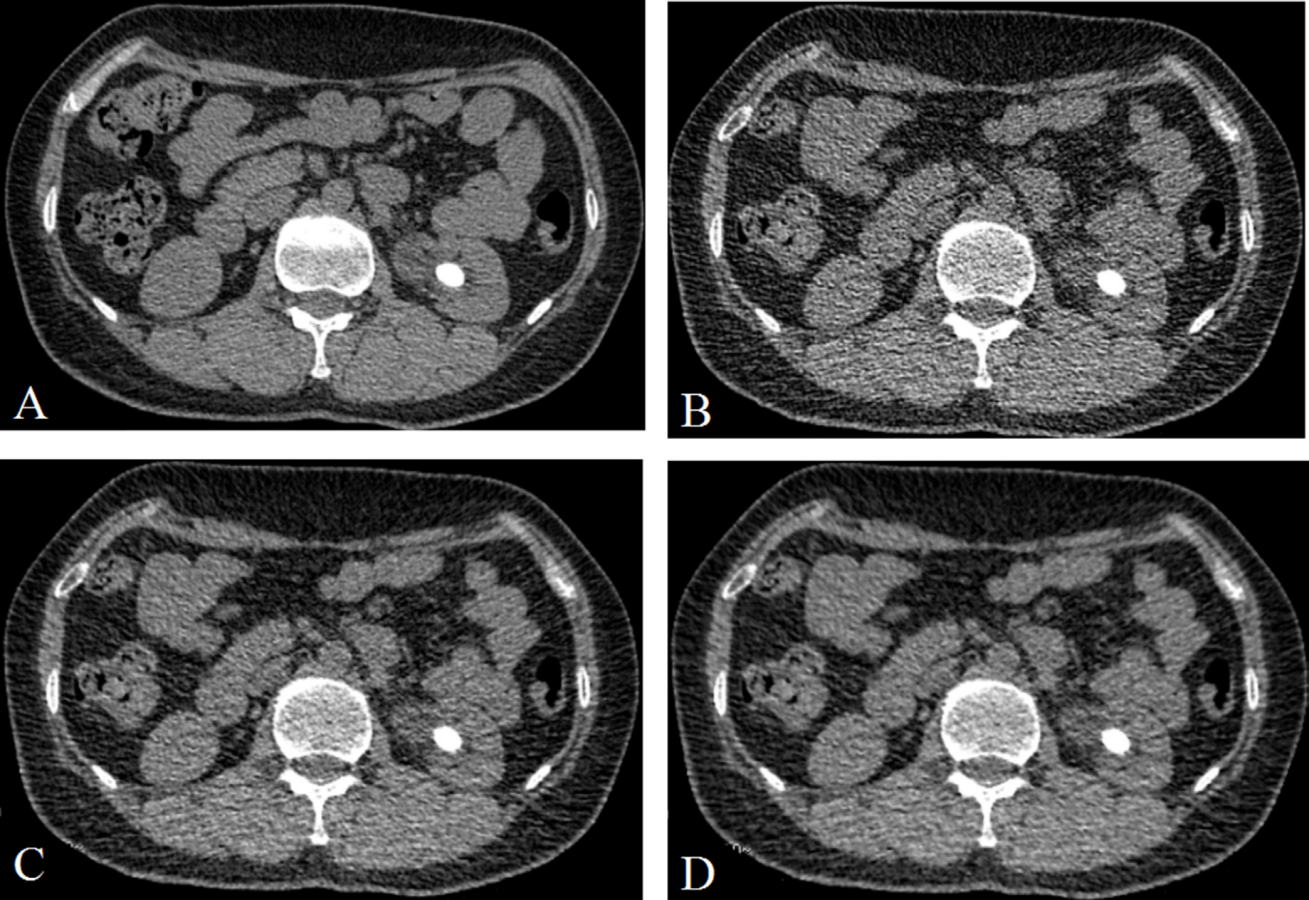
A 48 years old man with a stone (14.2 mm × 10.3 mm × 13.1 mm) in the left kindy (**A**) CDCT images, reconstructed with FBP; (**B**) LDCT image, reconstructed with FBP; (**C**) LDCT image, reconstructed with 60% ASIR; (**D**) LDCT images, reconstructed with 80% ASIR. The stone size were no obvious difference between the A and D.

The CDCT images were assessed to set the reference for the number, location, size, and the distance from stone to skin [SSD] of the stones. With LDCT image 347 stones were diagnosed. 5 missed stones concerned stones with a size below 1 mm located in the ureter. The stones size (lenggth, width, height) and the SSD were showed in Table 1. No significant differences were found between the CDCT and the LDCT in the size of stones (Figure 1) and the SSD (*P*-values = 0.689, 0.412, 0.107, and 0.183, respectively) among 347 stones. Two readers in the evaluation of the position and shape of stones in the LDCT and CDCT groups groups are basically the same.

**Table 1:** Stone characteristics as measured by CDCT and LDCT

Variable	CDCT	LDCT	*p*-value
Length (mm)	8.54 ± 7.75	8.52 ± 7.72	0.689
Width (mm)	5.73 ± 4.90	5.71 ± 4.87	0.412
Height (mm)	9.08 ± 8.52	9.03 ± 8.56	0.107
SSD (mm)	84.87 ± 25.95	85.06 ± 25.84	0.183

Abbreviations: CDCT, conventional dose computed tomography; LDCT, low dose computed tomography; SSD, the distance from stone to skin. Values are presented as mean ± standard deviation. All *p*-values were determined by paired *t*-test.

**Figure 2:**
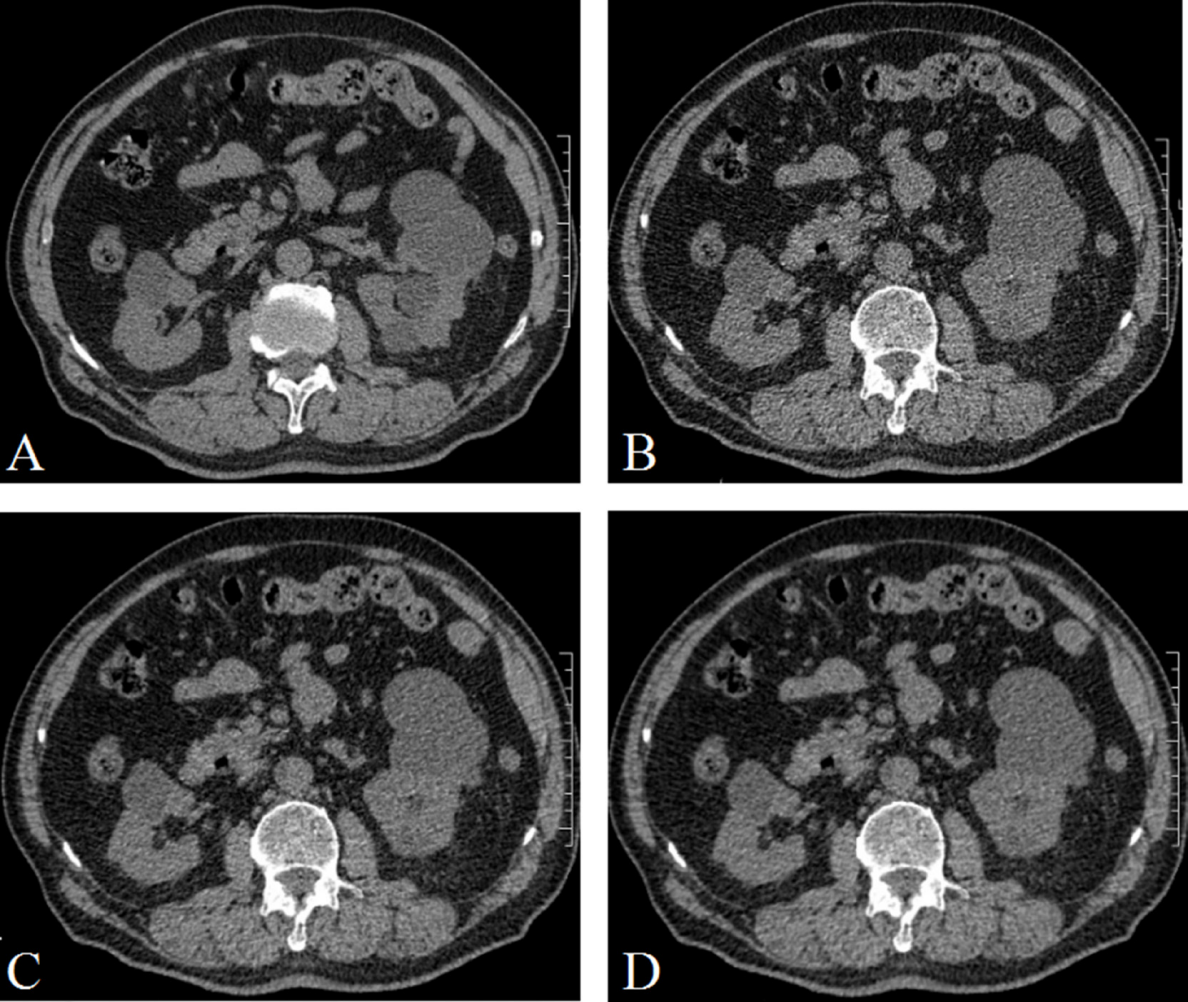
A 71 years old woman with several cyst in the renal (**A**) CDCT images, reconstructed with FBP; (**B**) LDCT image, reconstructed with FBP; (**C**) LDCT image, reconstructed with 60% ASIR; (**D**) LDCT images, reconstructed with 80% ASIR. The image quality was poor in B, and the image quality were similar in A and D.

Some other diseases were found as followed: 52 patients had hydronephrosis as a subsequent disease, 10 patients had renal cysts (Figure 2), 7 patients had liver cyst, 6 patients had gallstone, and one patients had perinephric abscess. Inter-observer agreement between the two readers was substantial (κ-value = 0.65–0.73).

The DLP and ED were recorded as an radiation dose. The average DLP and ED were 408.16 ± 119.04 mGy and 6.12 ± 1.79 mSv in CDCT, and were 138.19 ± 76.87 mGy and 2.07 ± 1.15 mSv in LDCT. Compared to CDCT, the radiation dose was reduced 66.1% in LDCT (*p* < 0.01).

**Figure 3:**
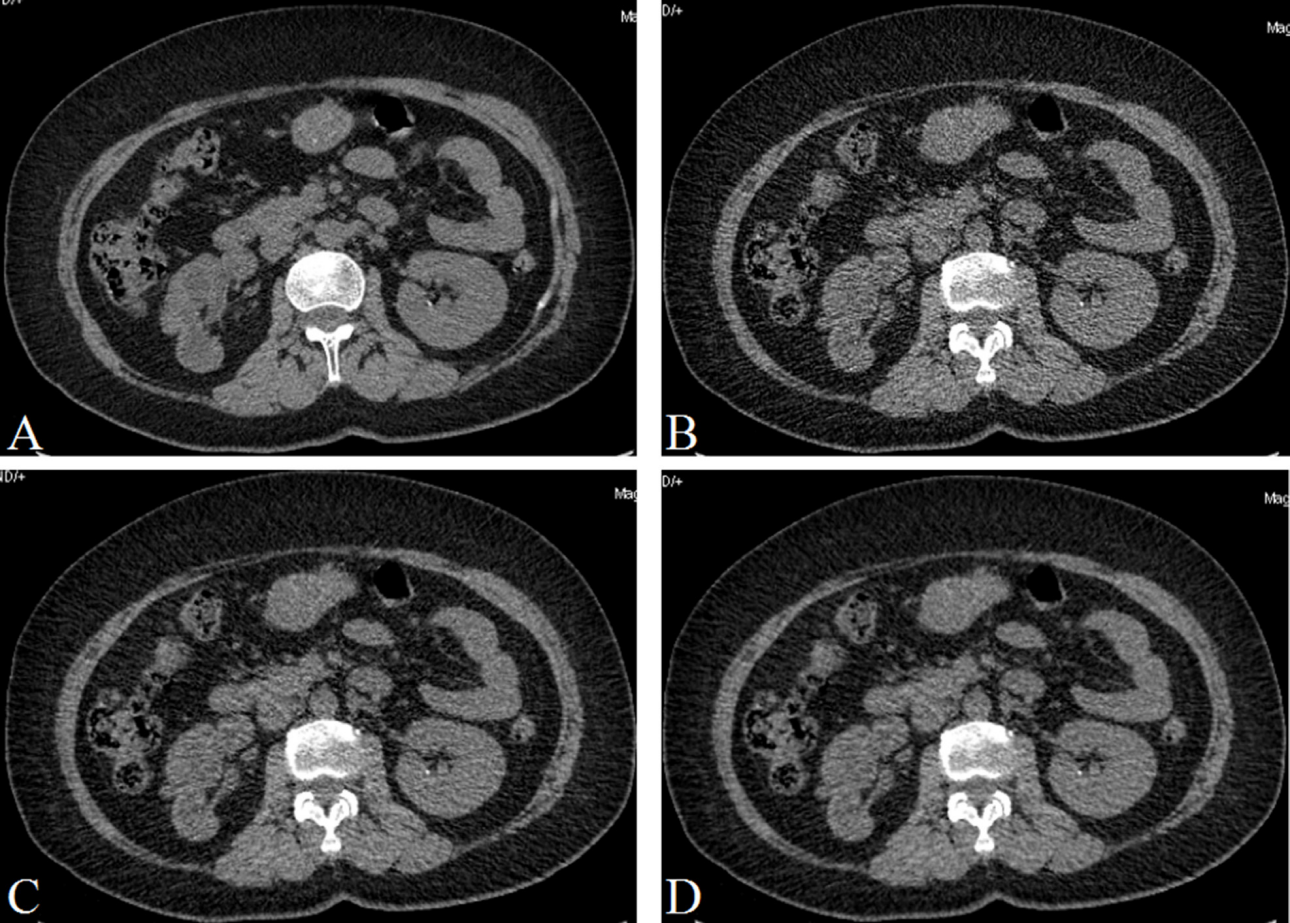
A 50 years old man with a small stone (1.8 mm × 1.6 mm × 1.7 mm) in the left renal (**A**) CDCT images, reconstructed with FBP; (**B**) LDCT image, reconstructed with FBP; (**C**) LDCT image, reconstructed with 60% ASIR; (**D**) LDCT images, reconstructed with 80% ASIR. The image quality was poor in B, and the image quality were similar in A and D.

The CDCT images were reconstructed with FBP and the LDCT images were reconstructed with FBP, 40% ASIR, 60% ASIR and 80% ASIR, repectively. The scores of image quality, image noise and diagnostic confidence were showed in Table 2. The LDCT images reconstructed with FBP had a poor image quality (mean score, 2.77), which was lower than the CDCT images reconstructed with FBP (mean score, 4.17) and the LDCT images reconstructed with 80% ASIR (mean score, 4.09). There were no significant difference between the CDCT images reconstructed with FBP and the LDCT images reconstructed with 80% ASIR with regard to the image quality (*P* = 0.229) (Figure 3).

**Table 2:** Comparison of image quality, image noise, and diagnostic confidence between the CDCT image and the LDCT image

Group	CDC FBP	LDCT FBP	LDCT 40% ASIR	LDCT 60% ASIR	LDCT 80% ASIR	*P*-value
Image quality	4.17	2.77	3.60	3.96	4.09	0.229
Image noise	1.03	2.27	1.59	1.17	1.09	0.147
Diagnostic confidence	3.00	1.96	2.74	2.91	2.94	0.043

Abbreviations: CDCT, conventional dose computed tomography; LDCT, low dose computed tomography; FBP, filtered back projectionl; ASIR, adaptive statistical iterative reconstruction. Values are presented as mean. All *p*-values were determined by Mann-Whitney *U* test.

The standard deviation of the mean CT number was measured as a objective image noise. The images noise was lowest on LDCT-80% ASIR images, which was similar to CDCT-FBP images (*p* = 0.015, 0.088, 0.087, respectively), and was highest on LDCT-FBP images. The result was showed in Table 3.

**Table 3:** Comparison of objective image noise between the CDCT image and the LDCT image

Location	CDCT FBP	LDCT FBP	LDCT 40% ASIR	LDCT 60% ASIR	LDCT 80% ASIR	*P*-value^*^
Fat	25.09 ± 3.60	42.03 ± 6.63	33.01 ± 5.72	28.85 ± 4.85	23.89 ± 3.94	0.015
Liver	29.67 ± 3.92	54.09 ± 7.77	41.21 ± 6.39	34.61 ± 5.64	28.54 ± 4.22	0.088
Muscle	28.13 ± 3.57	51.92 ± 8.39	38.88 ± 6.51	33.28 ± 5.62	27.16 ± 3.93	0.087

Abbreviations: CDCT, conventional dose computed tomography; LDCT, low dose computed tomography; FBP, filtered back projectionl; ASIR, adaptive statistical iterative reconstruction. Values are presented as mean ± standard deviation. All *p*-values were determined by paired *t*-test. ^*^:the *p*-values were derived from CDCT-FBP vs LDCT-80% ASIR.

## DISCUSSION

In recent years, conventional dose CT has become an indispensable tool for diagnosis of urinary calculi, which has high sensitivity and specificity of 94%–100% and 97%, respectively [15]. Compared to normal abdominal radiography, the evidently higher radiation dose of CDCT restrict its clinical application. Furthermore, the increasing incidence and recurrence of urinary calculi would lead to more than once CT examinations during the patients' lifetime. Recent studies have shown that exposure to radiation can lead to cancer, which has unpredictable and random effects [16]. Therefore, LDCT seems to become a preferable choice for the patients with urinary stones. However, its disadvantage includes the increased image noise and the reduced diagnostic confidence. Our study showed that LDCT, combined with ASIR, can reduce the radiation dose while maintain image quality.

Compared to the CDCT, the dose was reduced 66.1% in the LDCT. With the use of ASIR, the image noise was reduced, and the image quality was improved. The finding was consistent with prior studies. May et al. found that with the use of IRT, the dose reductions of up to 50% are available, while the image quality is retained [17].

Traditionally, FBP reconstruction technique serves as a principal method to improve image quality, but no substantially reduced radiation dose has been a limitation to this technique. If the radiation dose reduced widely, the image quality would beome poorer, because is unable to consistently generate diagnostic quality images with reduced x-ray tube currents (mA). Iterative reconstruction promises minimized image noise while allowing remarkable reduction of radiation dose exposure to patients. One type of iterative reconstruction technique, Adaptive statistical iterative reconstruction (ASIR), uses information obtained from the FBP algorithm as an initial building block for image reconstruction, which system is superior to the FBP in terms of noise and artifact reduction, since FBP does not take into account certain system hardware details (such as actual focal spot, detector sizes, and location) and system noise. ASIR is the iterative reconstruction techniques model the statistical behavior of measurements such as photon statistics and electronic noise, and also model the detector shape and size by modeling the detector response to incident photons, which can model the statistical variations in the distribution of the image noise to improve signal-to-noise ratio, while preserving the image contrast [18, 19].

At the same time, ASIR as a new image reconstruction technique, has been shown to improve image quality on LDCT images and reduce image noise [18–20]. Use a combination of ASIR, dose reduction does not affect stone characteristics, such as stone size and the SSD, which is important to make an treatment option [19]. As we known, stones with a diameter less than 5 mm have a 68% probability of passing, so stones with a diameter of 5 mm or less are often disposed conservatively [21]. What's more, large stones (> 3 mm) are easy to detect, but it is difficult to detect small stones (< 3 mm), by using CT at lower radiation doses [19, 22]. Poletti et al. [23] reported that LDCT showed a perfect sensitivity and specificity (96% and 100%, respectively) for the evaluation of urinary stones (≥ 3 mm). Our study show that the detection rate of large stones is consistent between LDCT images and CDCT images, the stone position and shape of stones in the LDCT and CDCT groups groups are basically the same in the Table 1. But in this study LDCT groups 5 missed stones concerned stones with a size below 1 mm located in the ureter. This may be due to the stone is too small, relative to the image noise can not be accurately identified. However, stones with a diameter less than 1 mm in ureter have a 100% probability of passing. In general, low-dose CT in the diagnosis of urinary stones, there is a greater clinical value.

Some prior studies showed that an ASIR percentage of 20%–60% has been found to be best for image quality and noise distribution [24]. Kulkarni et al. [25] showed that images from both 60% and 80% ASIR techniques performed in the LDCT group were better than FBP images, and there were no obvious differences between 60% and 80% ASIR images. McLaughlin et al. [26] showed that 70% ASIR LDCT images had higher diagnostic acceptability than 90% ASIR LDCT images. In our study, we choose 40%, 60% and 80% ASIR, and found that the image quality is the best in 80% ASIR. The scores of subjective image quality and image noise were similar between the CDCT and the LDCT. Notably, the scores of objective image noise in the LDCT were even lower than that in the CDCT.

In our study, the automatic tube current modulation has been used to reduce radiation dose. Noise index is one of the main parameters of the preset. With the noise index raising, the image noise levels would become high, while radiation dose will be low. We use “fixed noise index” in our study, the tube current will change from 10 mAs to 400 mAs according to the patients BMI. Tube current always reached the set maximum in the heavy patients, so these patients need to receive a higher radiation dose. Kulkarni et al. reported the similar result [25].

Our study also had some limitations. First, our study examined the patients using both CDCT and LDCT scanning. Previous studies used follow-up CT scan as a reference diagnosis, but there is a potential problem that the location of the stones will change during the process of the follow-up. We used CDCT images as an reference diagnosis, which can more precisely reflect the diagnostic performance. However, the special design made patients accept additional radiation exposure, so we need to made our best to reduce radiation exposures in follow-up studies. Only patients who fully agreed to participate in the experiments were involved in this study. Second, our sample size was not big enough, we are going on to enlarge the sample size in the future study. Third, the ingredients of the urianry stone could impact the image of CT. Because most of the patients in this study were diagnosed with small stones and did not undergo surgical treatment, it was not possible to further accurately evaluate the effect of stone composition on CT images.

Finally, we did not design scanning plan according to patients characteristic, such as the BMI of patients, so we need to improve and optimize our design in the future.

## MATERIALS AND METHODS

### Participants

This study was approved by the Institutional Review Board of the First Affiliated Hospital of Anhui Medical University, (Hefei, China), and an informed consent was obtained from all the patients after providing the study details, including information on the additional radiation dose.

This study is based on the Chinese. The study was performed on consecutive consenting adult patients undergoing CT scan for suspected or known kidney stone between September 2014 and March 2015, Subjects were eligible if they were fully agreed to participate in the experiments and capable of providing written informed consent were involved in this study. Exclusion criteria included pregnancy, age younger than 18 years, urinary tract abnormalities (medullary sponge kidney and ingle kidney), and kidney surgery, (a history of implanted device and malignancies) which could affect the quality of the results [14]. Gender, age, height and weight of all patients were recorded.

### CT technique

The examinations were performed with a 64-section multidetector CT scanner (Discovery CT750 HD; GE Healthcare), Automatic tube current modulation scanning was used (a tube current range of 10–400 mA), with noise index setting of 13 in CDCT and 25 in LDCT, and some other scanning parameters were as follows: gantry rotation time, 0.5 second; section thickness, 5 mm; and pitch, 1.375. Patients were placed in the supine position, scanning range from T12 vertebra to the edge of pubic symphysis.

### Image reconstruction

For all patients, the operating technologist reconstructed images on the scanner console immediately after the completion of CT examinations. The CDCT images were reconstructed by filtered back projection (FBP) and the LDCT images were reconstructed by both FBP and different settings of ASIR (40%, 60%, 80%). Images were reconstructed in the Axial and coronal planes by using a 1.25 mm section thickness. All the image data sets were then transmitted to the picture archiving and communication system for interpretation.

### Image analysis

**Figure 4:**
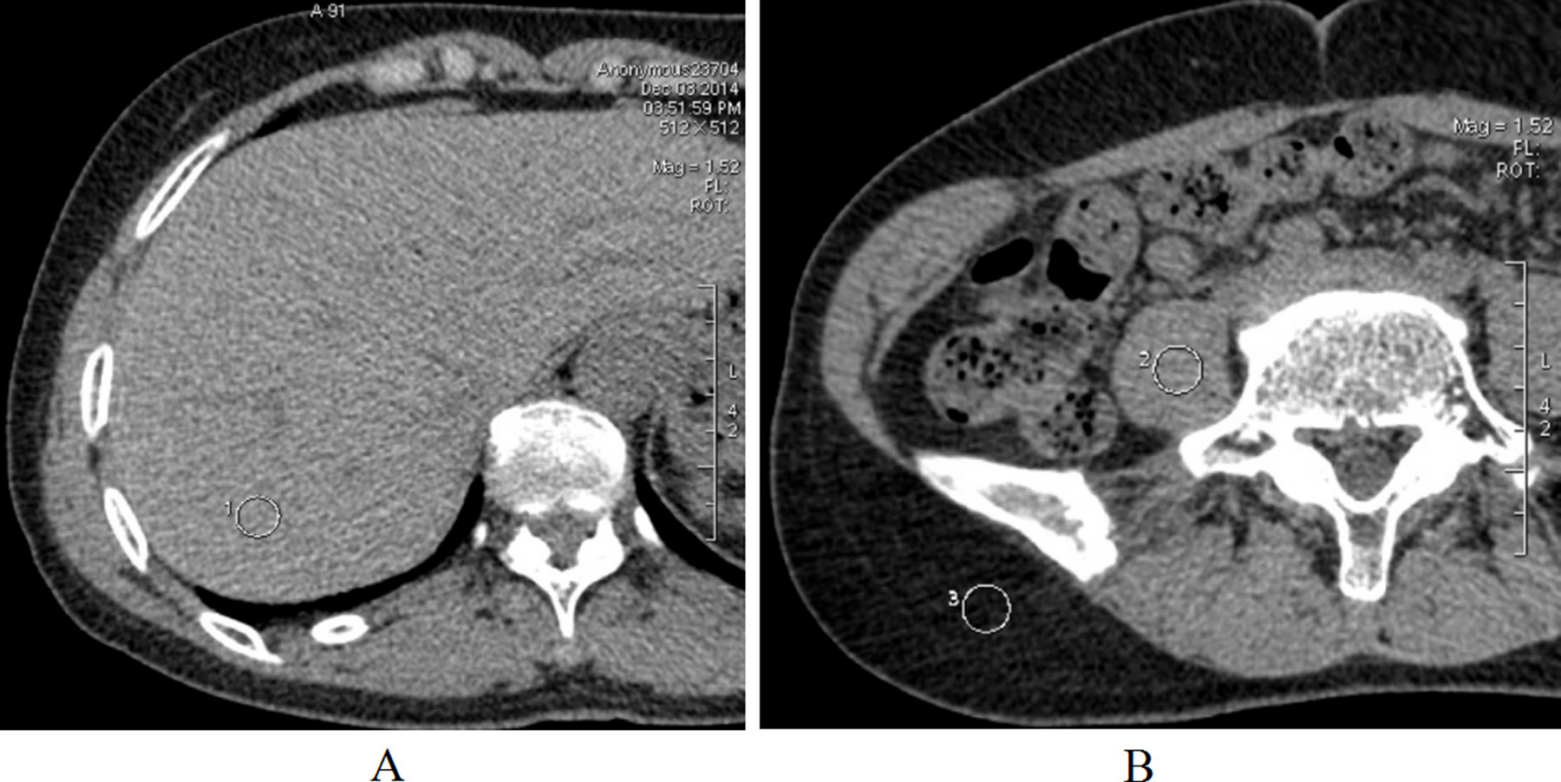
ROI 1 (**A**) in the right lobe of the liver, ROI 2 and 3 (**B**) in the paoas muscle, and the subcutaneous fat. Abbreviations: ROI, region of interest.

All images were randomized review by two experienced radiologists (with 8 and 6 years of experience in diagnosis of abdominal CT, respectively) independently. The standard deviation of the mean CT number was measured as the objective image noise, which was measured by placing a circular region of insterest of 80–100 mm^2^ in the paoas muscle, the right lobe of the liver, and the subcutaneous fat (Figure 4). Subjective image assessment included image quality, image noise, and diagnostic confidence. A score was derived for axial and coronal images with a soft-tissue window setting (width, 400 HU; level, 50 HU) on PACS system. The image quality was rated on a 5-point scale, (score1, poor image quality—not diagnostically acceptable for interpretation; score2, suboptimal image quality—worse-than-acceptable quality; score3, acceptable image quality—diagnostic interpretation possible; score 4, good image quality; and score 5, excellent image quality). A CT image that had an image-quality score of 3 or higher was considered acceptable for rendering an interpretation [25]. A subjective assessment of image noise and diagnostic confidence were rated on a 3-point scale (score 1, minimal; score 2, acceptable; and score 3, excessive—rendering diagnostic interpretation impossible) [25]. The readers also recorded the stone characteristics (number, location, size, and the distance from stone to skin [SSD]). The ureter was divided into three segment: the upper one-third of the ureter was from the renal pelvis to the superior border of the iliac crest; the middle one-third of the ureter was from the superior border of the iliac crest to the bottom of the sacroiliac joint; and the lower one-third of the ureter was from the bottom of the sacroiliac joint to the ureterovesical joint [27]. The radiation doses (mGy) were from the estimation of DLP that was generated by the CT scanner. The ED was calculated from the DLP by multiplying it by the conversion coefficient (0.015 mSv/mGy/cm).

### Statistical analysis

Statistical analysis was performed with SPSS16.0, all parametric variables were compared by using paired *t*-tests and Mann-Whitney *U* test between the two CT protocols, and statistical significance was indicated with a *p*-value < 0.05. The Cohen k test was used to assessment inter-observer agreement between the two readers (k of 0.19 or lower, poor; k of 0.20–0.39, fair; k of 0.40– 0.59, moderate; k of 0.60–0.79, substantial; k of 0.80–1.00, perfect.).

## CONCLUSIONS

In summary, LDCT is an effective method for diagnosing urinary stones, which can obviously reduce the radiation dose while maintain image quality comparable to CDCT.
